# Molecular epidemiology of human sporotrichosis in Venezuela reveals high frequency of *Sporothrix globosa*

**DOI:** 10.1186/s12879-015-0839-6

**Published:** 2015-02-25

**Authors:** Emma Camacho, Isabel León-Navarro, Sabrina Rodríguez-Brito, Mireya Mendoza, Gustavo A Niño-Vega

**Affiliations:** Centro de Microbiología y Biología Celular, Instituto Venezolano de Investigaciones Científicas, Caracas, Venezuela; Laboratorio de Micología, Instituto de Biomedicina, Caracas, Venezuela

**Keywords:** *Sporothrix schenckii* species complex, *Sporothrix globosa*, Sporotrichosis, Clinical forms, Phenotypic identification, Phylogenetic analysis, American continent, Venezuela

## Abstract

**Background:**

Sporotrichosis is a cutaneous and subcutaneous fungal disease of humans and other mammals, known to be caused by the *Sporothrix schenckii* species complex, which comprises four species of clinical importance: *S. brasiliensis*, *S. globosa*, *S. luriei*, and *S. schenckii sensu stricto*. Of them, *S. globosa* and *S. schenckii s. str.* show global distribution and differences in global frequency as causal agents of the disease. In the Americas, only three species are present: *S. schenckii s. str.*, *S. brasiliensis* (so far, only reported in Brazil), and *S. globosa*. In Venezuela, since the first case of sporotrichosis reported in 1935, *S. schenckii* have been considered its unique etiological agent. In the present work, the presence of more than one species in the country was evaluated.

**Methods:**

By phenotypic key features and molecular phylogeny analyses, we re-examined 30 isolates from diverse Venezuelan regions belonging to the fungi collection of Instituto de Biomedicina, Caracas, Venezuela, and national reference center for skin diseases. All isolates were collected between 1973 and 2013, and maintained in distilled water.

**Results:**

Sporotrichosis in Venezuela is mainly caused by *S. schenckii s. str.* (70%). However, a significant proportion (30%) of sporotrichosis cases in the country can be attributable to *S. globosa*. A correlation between intraspecific genotypes and clinical presentation is proposed.

**Conclusions:**

Our data suggest that sporotrichosis various clinical forms might be related to genetic diversity of isolates, and possibly, to diverse virulence profiles previously reported in the *S. schenckii* species complex. *Sporothrix globosa* was found to be the causative agent of 30% of sporotrichosis for the Venezuelan cases re-examined, the highest frequency of this species so far reported in the Americas. The high genetic variability presented by *S. schenckii s. str.* indicates that species distinction based on phenotypic key features could be a challenging and uncertain task; molecular identification should be always employed.

## Background

Over the last few years, the *Sporothrix schenckii* species complex (formerly known as *S. schenckii*) has received special interest due to the increasing number of infections caused by it worldwide, particularly in immunocompromised patients [1], and epidemic outbreaks in cats in Brazil [2]. It is responsible for sporotrichosis, a chronic granulomatous subcutaneous mycosis of humans and other mammals. Primary infection has been reported as caused by traumatic inoculation of environmental material carrying saprophytic hyphae and conidia of the etiologic agents [2,3]. Once into the warm-blooded host, these dimorphic fungi are capable to convert into pathogenic yeasts, causing infections that range from fixed cutaneous localized lesions to severe disseminated sporotrichosis [3-5]. However, zoonotic transmission is also possible, by scratches or bites from asymptomatic or infected animals, with cats being the main vectors through which the disease is transmitted to humans or other animals [6-8]. The last makes direct inoculation of pathogenic yeast a plausible source of infection. Although the disease has a global occurrence, endemic areas are mostly located in tropical and subtropical countries [6,9]; furthermore, *Sporothrix* infections may take epidemic proportions [7,8,10] and its distinct etiological agents differ in virulence profiles [11-13], antifungal susceptibility [14,15], and geographic distribution [16].

Molecular phylogenetic analyses have led to description of at least four cryptic species of clinical relevance within the *S. schenckii* species complex, comprising *S. brasiliensis*, *S. schenckii sensu stricto* (*s. str*), *S. globosa* and *S. luriei* [2,8,17-19]. Scattered reports of *Sporothrix* spp. outside these four clades, causing clinical cases have been published, such as *S. mexicana* [16,20] and *S. pallida* [21], or by close relatives in the genus *Ophiostoma*, i.e. *O. piceae* [22] and *O. stenoceras* [23]. However, these species appear to lack human-pathogenic potential, thus they are proposed to be accidentally pathogenic [17]. Besides molecular phylogeny, morphological and physiological key features have been proposed for species recognition within the *Sporothrix* species complex [18,19].

In Venezuela, sporotrichosis is the second most common subcutaneous mycosis after chromomycosis. It was first described in 1935, nevertheless the exact prevalence in the country is unknown [24]. According to conventional mycological procedures, as well as epidemiological data, all isolates related to sporotrichosis have been identified as *Sporothrix schenckii*.

The aim of the present study was to determine if, besides *S. schenckii*, other *Sporothrix* species of the complex might be present in the country. We re-examined 30 isolates, by phenotypic and molecular methods, discovering that *S. globosa* was the etiological agent in one third of the cases.

## Methods

### Fungal isolates and strains used

Thirty isolates (29 clinical and 1 environmental) from different Venezuelan regions (Coastal range; *n* = 22, The Andes; *n* = 7; The Plains; *n* = 2) were examined in this study (Table 1). All isolates were previously identified as *S. schenckii* by means of morphology (macro and microscopic studies), ability of isolates to reverse to yeast-like cells at 37°C, and serological tests to patients from where they were isolated, all performed in the Mycology Laboratory at Instituto de Biomedicina, Caracas, Venezuela, a national reference center for skin diseases. Isolates were taken as part of standard patient care, and no ethical approval was required for their use. They have been kept as part of the laboratory fungal collection over a period of 40 years (from 1973 to 2013) and were selected for inclusion in this study based on their geographic distribution. They were preserved in distilled water (Castellani’s method), and recovered by growth on Sabouraud dextrose agar (SDA) complemented with 150 μg ml^−1^chloramphenicol at room temperature during 7 days. As reference, strains *S. schenckii s. str.* ATCC-MYA 4820, and *S. brasiliensis* ATCC-MYA 4823 (provided by Dr. L. Bezerra, Universidade do Estado do Rio de Janeiro, Brazil), as well as *S. pallida* CBS 302.73^T^, *S. globosa* FMR 9023, and *S. mexicana* FMR 9108 kindly provided by Dr. J. Cano (Reus, Spain) were used.

**Table 1:** **Isolates used in this study**

**Isolate**	**Species (by molecular genotyping)**	**Source**	**Origin**	**GenBank Accession N°**	
				**CAL**	**ITS**
C0014	*S. schenckii*	Clinical, Human (lymphocutaneous)	Coastal range (Distrito Capital, Venezuela)	^1^KF478909	^1^KJ999877
C0329	*S. globosa*	Clinical, Human (fixed cutaneous)	The Andes (Táchira, Venezuela)	^1^KF478892	^1^KJ999878
C0596	*S. schenckii*	Clinical, Human (fixed cutaneous)	Coastal range (Miranda, Venezuela	^1^KF478896	^1^KJ999879
C1163	*S. schenckii*	Clinical, Human (lymphocutaneous)	Coastal range (Distrito Capital, Venezuela)	^1^KF478910	^1^KJ999880
C2606	*S. schenckii*	NA	Coastal range (Distrito Capital, Venezuela)	^1^KF478887	^1^KJ999881
C2656	*S. schenckii*	Clinical, Human (fixed cutaneous)	The Andes (Trujillo, Venezuela)	^1^KF478897	^1^KJ999882
C2745	*S. schenckii*	Clinical, Human (fixed cutaneous)	The Plains (Monagas, Venezuela)	^1^KF478891	^1^KJ999883
C2888	*S. schenckii*	Clinical, Human (lymphocutaneous)	Coastal range (Distrito Capital, Venezuela)	^1^KF478886	^1^KJ999884
C3037	*S. schenckii*	Clinical, Human (disseminated)	Coastal range (Distrito Capital, Venezuela)	^1^KF478888	^1^KJ999885
C3527	*S. schenckii*	NA	Coastal range (Distrito Capital, Venezuela)	^1^KF478898	^1^KJ999886
C3673	*S. globosa*	Clinical, Human (fixed cutaneous)	Coastal range (Distrito Capital, Venezuela)	^1^KF478892	^1^KJ999887
C3705	*S. schenckii*	Clinical, Human (lymphocutaneous)	Coastal range (Distrito Capital, Venezuela)	^1^KF478884	^1^KJ999888
C4346	*S. schenckii*	Clinical, Human (lymphocutaneous)	Coastal range (Distrito Capital, Venezuela)	^1^KF478889	^1^KJ999889
C5000	*S. schenckii*	Clinical, Human (lymphocutaneous)	Coastal range (Aragua, Venezuela)	^1^KF478885	^1^KJ999890
C5859	*S. globosa*	Clinical, Human (fixed cutaneous)	Coastal range (Vargas, Venezuela)	^1^KF478893	^1^KJ999891
C7012	*S. schenckii*	Clinical, Human (lymphocutaneous)	The Andes (Táchira, Venezuela)	^1^KF478899	^1^KJ999892
C8213	*O. stenoceras*	Clinical, Human (fixed cutaneous)	Coastal range (Distrito Capital, Venezuela)	^1^KF478913	^1^KJ999893
C8287	*S. schenckii*	Clinical, Human (fixed cutaneous)	Coastal range (Distrito Capital, Venezuela)	^1^KF478900	^1^KJ999894
C8704	*S. schenckii*	NA	Coastal range (Distrito Capital, Venezuela)	^1^KF478890	^1^KJ999895
C8775	*S. globosa*	Clinical, Human (fixed cutaneous)	Coastal range (Aragua, Venezuela)	^1^KF478901	^1^KJ999896
C8857	*S. globosa*	Clinical, Human (fixed cutaneous)	The Andes (Trujillo, Venezuela)	^1^KF478902	^1^KJ999897
C8888	*S. schenckii*	Clinical, Human (lymphocutaneous)	The Andes (Trujillo, Venezuela)	^1^KF478903	^1^KJ999898
C8962	*S. globosa*	Clinical, Human (fixed cutaneous)	The Plains (Cojedes, Venezuela)	^1^KF478904	^1^KJ999899
C8981	*S. globosa*	Clinical, Human (fixed cutaneous)	The Andes (Mérida, Venezuela)	^1^KF478905	^1^KJ999900
C9254	*S. globosa*	Clinical, Human (fixed cutaneous)	Coastal range (Distrito Capital, Venezuela)	^1^KF478911	^1^KJ999901
C9300	*S. schenckii*	Clinical, Human (lymphocutaneous)	The Andes (Trujillo, Venezuela)	^1^KF478906	^1^KJ999902
C9526	*S. schenckii*	NA	Coastal range (Distrito Capital, Venezuela)	^1^KF478907	^1^KJ999903
C9862	*S. schenckii*	Clinical, Human (fixed cutaneous)	Coastal range (Distrito Capital, Venezuela)	^1^KF478912	^1^KJ999904
C9887	*S. globosa*	Clinical, Human (fixed cutaneous)	Coastal range (Distrito Capital, Venezuela)	^1^KF478908	^1^KJ999905
A0001	*S. schenckii*	Environmental, Soil	Coastal range (Aragua, Venezuela)	^1^KF478894	^1^KJ999876
**ATCC MYA-4820**	***S. schenckii***	**Clinical, Human**	**Brazil**	^**2**^ **JF313361**	^**3**^ **JQ070111**
**ATCC MYA-4823**	***S. brasiliensis***	**Feline sporothricosis**	**Brazil**	^**2**^ **JF313351**	^**3**^ **JQ070114**
**CBS 302.73** ^**T**^	***S. pallida***	**Environmental**	**United Kingdom**	^**4**^ **AM398396**	^**1**^ **KJ999906**
**FMR 9023**	***S. globosa***	**Clinical, Human**	**Japan**	^**4**^ **AM398393**	^**1**^ **KJ999907**
**FMR 9108**	***S. mexicana***	**Environmental**	**Mexico**	^**4**^ **AM398393**	^**5**^ **FN549906**

Previously genotyped strains used as reference, in bold.

^1^This study; ^2^Teixeira, M. and Lopes-Bezerra, L.M., direct submission; ^3^Suh, S.-O. and Zhou, J.J, direct submission; ^4^ATCC Mycology Authentication Project.

^5^Madrid *et al*. [33]. NA, data non-available.

### Phenotypic characterization

Isolates were phenotypically identified according to Marimon *et al*. [18]. Macroscopic features of colonies were studied by culturing isolates on potato dextrose agar (PDA, HIMEDIA, India) plates, incubated at room temperature (growth control), 30°C and 37°C in dark. Petri dishes (90 × 15 mm, with 8 mm PDA) were centrally inoculated with portions of the colonies of the fungi, approximately 1 mm in diameter, and incubated upside down. After 14 and 21 days, colony diameters (in millimeters) were measured in duplicate for growth temperatures of 30°C and 37°C, and the mean of the recorded measured diameters taken for final analysis. Microscopic features of conidia were found to be the same for reference strains and a sample of Venezuelan isolates when lactrimel agar (LA) was used instead of the corn meal agar (CMA) used by Marimon et al. [18]. So for the rest of the study, microscopic features of conidia were determined from slide cultures made on lactrimel agar (10 g honey, 20 g wheat flour, 200 ml milk, 9.5 g agar, 800 ml distilled water and 150 μg ml^−1^ chloramphenicol) after 12–15 days of incubation in the dark in a humid chamber. Coverslips were mounted in lactophenol cotton blue (0.05% (w/v)), 2% phenol, 2% lactic acid and 4% glycerin), and examined under a light microscope (Leica DM2000) at 40X and 100X. Carbohydrate assimilation tests for sucrose and raffinose were conducted in 96-well microplates, using 150-μl of a working solution composed of yeast nitrogen base (YNB) medium (Sigma-Aldrich, USA) with corresponding test sugar, or plain (only base medium), as described by Marimon et al. [18]. Briefly, the isolates were grown on potato dextrose broth for 7 days at 23°C, or on Brain-Heart Infusion agar (BHI, OXOID, England), at 30°C for isolates that did not sporulated with the previous conditions. Conidia were collected by filtering 10 to 20 ml liquid culture through sterile gauze and quantified in a spectrophotometer at 600 nm. Each suspension was adjusted to an optical density that ranged from 0.21 to 0.29, corresponding to a final inoculum in the microplate of 2×10^5^ to 2×10^6^ CFU/ml. Each well was inoculated with 50 μl inoculum and 150 μl YNB medium with corresponding carbohydrate, at a final concentration of 3.75%. YNB medium without sugar was used as negative control, and with glucose as positive control. Microplates were read at 5 and 10 days of incubation at 25°C, experiments were conducted by duplicate. The viability of the conidia was verified by plating 20 μl of a 1:500 dilution of each inoculum onto YPD (0.5% yeast, 0.5% peptone, 1.5% dextrose) plates. In case of discordant results, experiments were repeated at least two additional times on different days.

### Molecular characterization

Total genomic DNA was isolated from 3-day-old mycelial cultures grown on YPD broth, following the protocol described in [25]. Partial amplification of the calmodulin (CAL) locus was obtained by using degenerated primers CL1 and CL2A [26], amplifying a region corresponding to exons 3 through 5 of the CAL gene. ITS regions were amplified from genomic DNA with universal primers ITS5 and ITS4 as described by Zhou et al. [17]. Following PCR, amplicons were purified with the CONCERT™ Rapid PCR purification system (Life Technologies, USA) and sent for sequencing on both strands to Macrogen Inc (Seoul, Korea), with the same primers used for PCR. Assembly of the sequences was done with the Contig program within the Vector NTI suite (Vector NTI, InforMax, Inc, USA). Homology searches were performed on GenBank database using BLAST [27]. The newly reported sequences generated in this study were deposited in the GenBank/EMBL/DDBJ database under accession numbers listed in Table 1.

### Phylogenetic analysis

**Figure 1:**
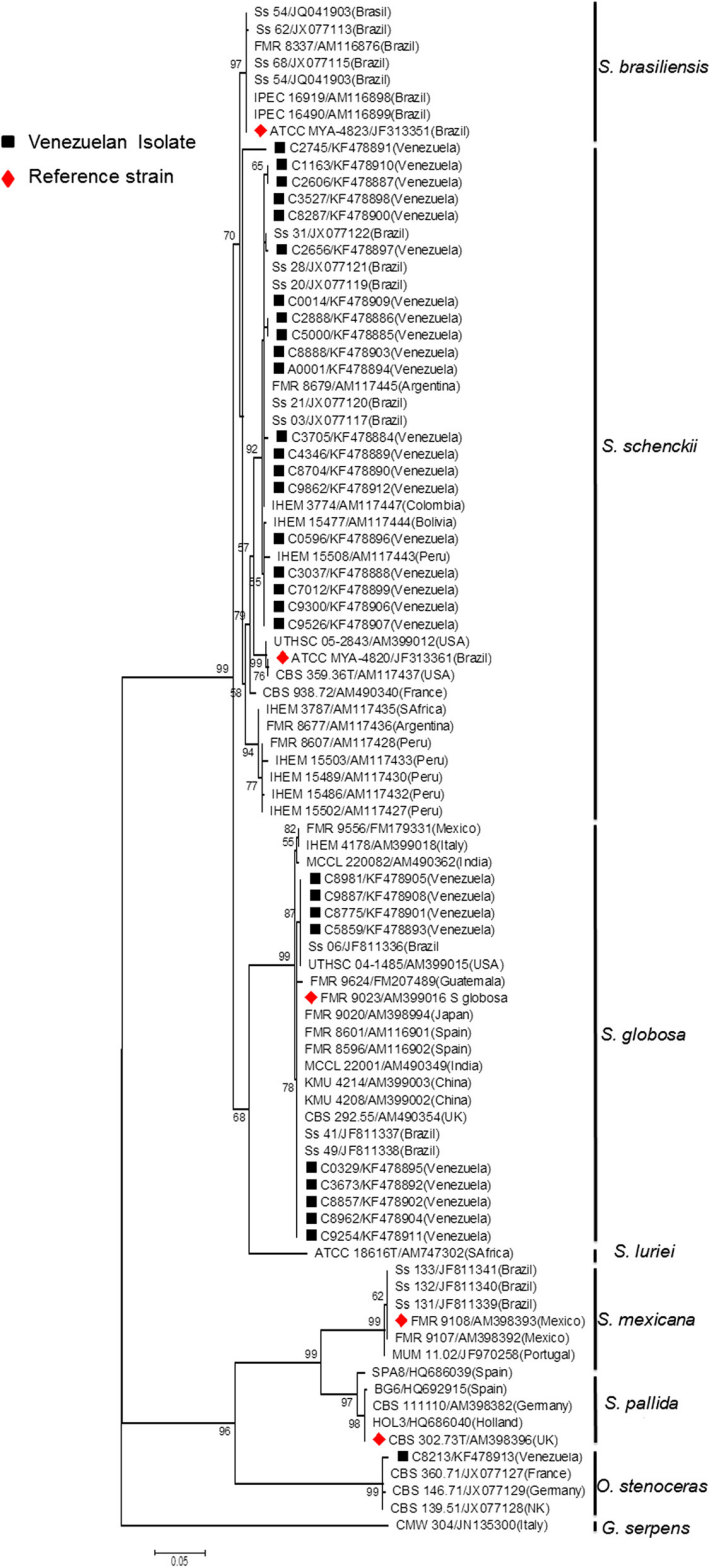
**Phylogenetic relationships of** ***Sporothrix schenckii*** **complex isolates, inferred from CAL sequences.** The phylogenetic tree was built with MEGA 5.1, by the Maximum Likelihood method based on the Kimura 2-parameter model. Thirty sequences from this study and sixty retrieved from previous studies were used for analysis. Number close to branches represents Bootstraps support values based on 1000 bootstrap replications. GenBank accessions numbers are indicated next to strain code in branch labels.

**Figure 2:**
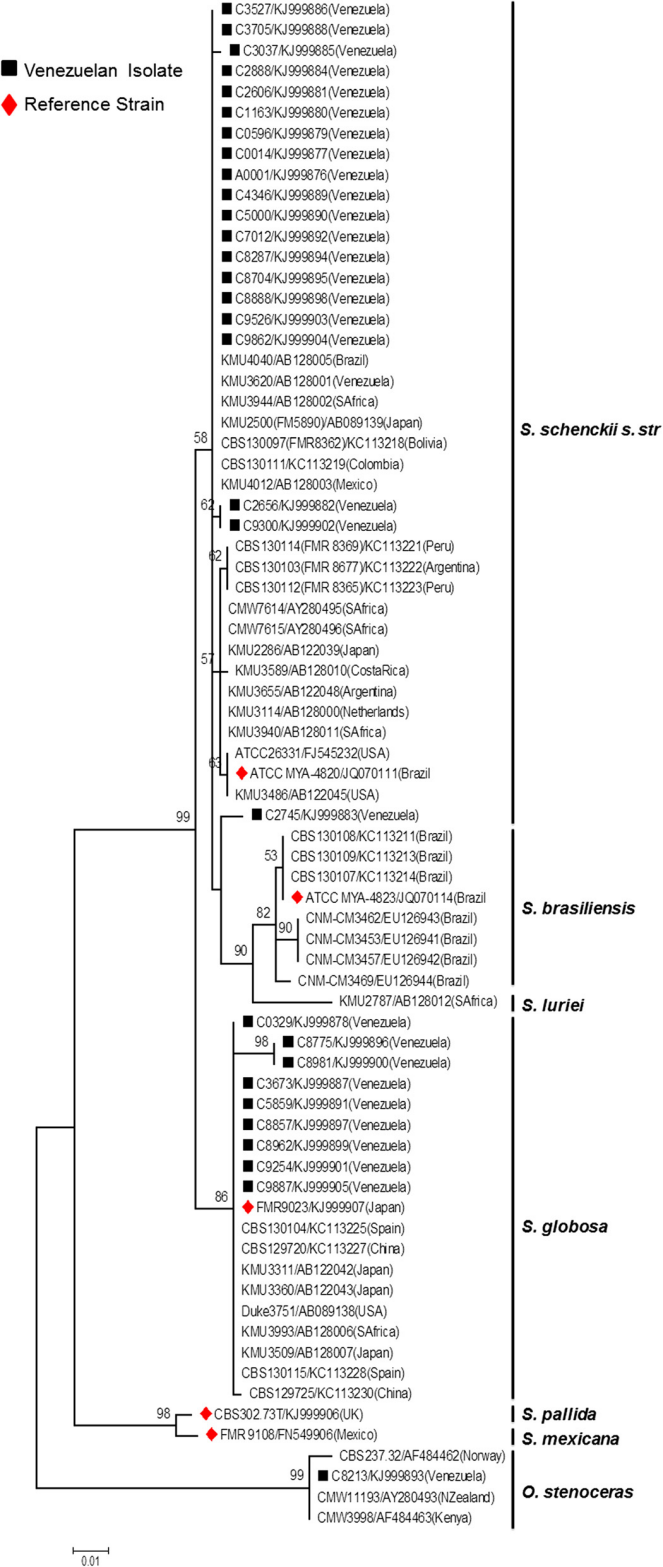
**Phylogenetic relationships of** ***Sporothrix schenckii*** **complex isolates inferred from ITS sequences.** The phylogenetic tree was built with MEGA 5.1, by the Maximum Likelihood method based on the Kimura 2-parameter model. Thirty sequences from this study and forty four from previous reports were used for analysis. Number close to branches represents Bootstraps support values based on 1000 bootstrap replications. GenBank accessions numbers are indicated next to strain code.

*Sporothrix*-calmudulin-related and *Sporothrix*-ITS-related sequences, previously reported to be obtained with the same two sets of primers here used, were retrieved from Genbank and included in the analyses (Figures 1 and 2) [16,18]. As an outgroup, the saprophytic fungus *Grosmannia serpens* (Ophiostomataceae) [28] was included for the CAL analysis. The multiple nucleotide sequence alignment was performed using the ClustalW algorithm implemented in MEGA5.2 software [29]. Evolutionary analyses were also conducted in MEGA5.2 as described by Rodrigues *et al*. [16].

### Genetic variation analysis

To evaluate genetic variation, the CAL locus was used as nuclear marker. Nucleotide (π) and haplotype (Hd) diversities [30] were estimated using DnaSP software, version 5.10.01 [31]. Sites containing gaps and missing data were not considered in the analysis.

## Results

A total of 30 previously identified *S. schenckii* Venezuelan isolates (29 clinical and 1 environmental) (Table 1) were re-examined on the basis of phenotypic key features (i.e. growth at various temperatures, macroscopic and microscopic features, and carbohydrate assimilation, according to Marimon *et al*. [18]), as well as molecular phylogeny inferred from CAL and ITS sequences. Based on the result obtained for reference strain *S. globosa* FMR 9023 (Table 2), restricted growth at 37°C was set at ≤ 6.4 mm colony diameter (maximum diameter reached in our study by this reference strain, previously identified by Marimon *et al*. [18]). Only 8 out of 30 isolates (26.67%) were clearly identified when the summary of key features for *Sporothrix* species differentiation, proposed by Marimon *et al*. [18], was strictly followed (Table 2).

**Table 2:** **Isolates characterized by phenotypic and molecular methods included in the study**

**Isolate**	**Mean colony diameter (mm) ± SD on PDA during 21 days**		**Assimilation test result**		**Morphology of dark sessile conidia**	**Identification on physiological tets**	**Identification on CAL and ITS sequences**
	**30°C**	**37°C**	**Sucrose**	**Raffinose**			
C0014	45.5 ± 0.7	12.0 ± 0.0	+	-	Triangular	NC	*S. schenckii*
C0329	46.5 ± 2.1	6.5 ± 0.7	+	-	Subglobose	NC	*S. globosa*
C0596	53.5 ± 0.7	23.0 ± 0.0	+	-	Triangular	NC	*S. schenckii*
C1163	52.5 ± 2.1	11.5 ± 0.7	+	+	Triangular	NC	*S. schenckii*
C2606	56.0 ± 0.0	11.5 ± 0.7	+	-	Triangular	NC	*S. schenckii*
C2656	52.5 ± 0.7	13.5 ± 0.7	+	-	Triangular	NC	*S. schenckii*
C2745	45.5 ± 0.7	10.0 ± 0.0	+	-	Triangular	NC	*S. schenckii*
C2888	53.5 ± 0.7	12.5 ± 0.7	+	-	Triangular	NC	*S. schenckii*
C3037	57.0 ± 1.4	15.0 ± 0.0	+	+	Triangular	NC	*S. schenckii*
C3527	54.0 ± 1.4	17.0 ± 1.4	+	+	Triangular	NC	*S. schenckii*
C3673	41.5 ± 0.7	5.5 ± 0.7	+	-	Globose	*S. globosa*	*S. globosa*
C3705	50.0 ± 1.4	17.0 ± 1.4	+	+	Triangular	*S. schenckii*	*S. schenckii*
C4346	52.0 ± 1.4	15.0 ± 0.0	+	+	Triangular	NC	*S. schenckii*
C5000	55.5 ± 0.7	16.5 ± 0.7	+	+	Triangular	NC	*S. schenckii*
C5859	38.0 ± 0.0	4.0 ± 0.0	+	-	Globose	*S. globosa*	*S. globosa*
C7012	58.0 ± 0.0	22.0 ± 0.0	+	+	Triangular	NC	*S. schenckii*
†C8213	25.5 ± 4.9	12.7 ± 3.1	+	-	NP	NC	*O. stenoceras*
†C8287	30.0 ± 0.0	3.0 ± 0.0	+	-	Triangular	*S. globosa*	*S. schenckii*
C8704	44.5 ± 0.7	7.0 ± 1.4	+	-	Triangular	NC	*S. schenckii*
†C8775	23.0 ± 0.0	3.0 ± 2.8	-	-	Subglobose	NC	*S. globosa*
C8857	33.5 ± 0.7	3.0 ± 2.8	+	-	Subglobose	*S. globosa*	*S. globosa*
C8888	52.5 ± 0.7	14.5 ± 0.7	+	-	Triangular	NC	*S. schenckii*
C8962	41.0 ± 0.0	5.5 ± 0.7	+	-	Subglobose	*S. globosa*	*S. globosa*
C8981	38.0 ± 0.0	3.0 ± 0.0	+	-	Globose	*S. globosa*	*S. globosa*
C9254	44.5 ± 0.7	8.0 ± 0.0	+	-	Globose	NC	*S. globosa*
C9300	44.5 ± 0.7	13.5 ± 0.7	+	-	Triangular	NC	*S. schenckii*
C9526	42.0 ± 0.0	13.5 ± 0.7	+	+	Triangular	*S. schenckii*	*S. schenckii*
C9862	52.5 ± 2.1	12.0 ± 1.4	+	-	Triangular	NC	*S. schenckii*
C9887	37.5 ± 0.7	3.5 ± 0.7	+	-	Globose	*S. globosa*	*S. globosa*
A0001	58.5 ± 0.7	15.5 ± 0.7	+	-	Triangular	NC	*S. schenckii*
**ATCC MYA-4820**	**41.0 ± ** **0.0**	**13.0** ** ± ** **1.4**	**+**	**+**	**Triangular**	***S. schenckii***	***S. schenckii***
**ATCC MYA-4823**	**43.5 ± ** **0.7**	**13.0** ** ± ** **1.4**	**-**	**-**	**Globose**	***S. brasiliensis***	***S. brasiliensis***
**CBS 302.73** ^**T**^	**76.5 ± ** **0.7**	**11.0** ** ± ** **0.0**	**+**	**-**	**NP**	***S. pallida***	***S. pallida***
**FMR 9023**	**33.5** ** ± ** **0.7**	**5.0** ** ± ** **1.4**	**+**	**-**	**Subglobose**	***S. globosa***	***S. globosa***
**FMR 9108**	**73.0** ** ± ** **1.4**	**16.5** ** ± ** **0.7**	**+**	**+**	**Ellipsoidal**	***S. mexicana***	***S. mexicana***

Reference strains in bold.

NC, non-conclusive; NP, not present; †Isolates showing atypical phenotypic profiles.

One isolate, *S. schenckii* C8287, was misidentified by following the phenotypic features solely, requiring molecular genotyping for proper identification; it was labeled as *S. globosa* due to its restrictive growth at 37°C, and positive/negative assimilation profile for sucrose and raffinose. The remaining 21 isolates (70.00%) showed inconclusive results, requiring molecular genotyping for proper identification (Table 2). The most ambiguous results (colony growth over 50 mm diameter at 30°C and/or negative assimilation of raffinose) were shown by *S. schenckii s. str.* isolates, for which just 2 out of 20 (10%) were properly identified without the need of molecular phylogeny analysis. However, it is important to notice that when the microscopic features of sessile conidia (pigmentation and morphology) were taken solely, correlation between identification by these features and molecular identification was found (Table 2).

Genotyping of the isolates was performed by using the amplified fragment from the CAL locus. The complete alignment included 90 sequences, 30 generated in this study and 60 retrieved from previous studies, respectively [16,18]. The aligned CAL sequences were 595 bp long, including 302 invariable characters, 239 variable parsimony-informative sites (40.17%) and 43 singletons. Twenty nine (29) of the 30 Venezuelan isolates clustered within the clinical clades (as defined by previous phylogenetic analyses of *Sporothrix* spp. based on CAL [6,16,32]). Of those 29 isolates, 20 (>60%) grouped as *S. schenckii s. str.*, while 9 (30%) grouped as *S. globosa* (Figure 1). One isolate (C8213), clustered within the environmental clades as *O. stenoceras* (Figure 1). To corroborate these results, a second phylogenetic analysis was performed, using ITS sequences generated by the universal primers ITS4 and ITS5. Seventy four (74) nucleotide sequences, 30 generated in this study and 44 previously reported [17] were aligned. The aligned ITS sequences were 506 bp long, including 407 invariable characters, 85 variable parsimony-informative sites (16.79%) and 12 singletons. The Venezuelan isolates were distributed in complete agreement with the CAL-generated phylogeny, with the Venezuelan isolates of *S. schenckii s. str.* and *S. globosa* (pathogenic species), and *O. stenoceras* (accidentally pathogenic species) showing strong, statistically supported separation (99% bootstrap) between clinical and environmental clades (Figure 2).

Assessment of the Venezuelan *Sporothrix* population genetic diversity was explored by using the DnaSP software [31] and CAL as nuclear marker. The haplotype analysis of calmodulin sequences (n = 65) divided the isolates into 17 Hap groups. A total of 15 and 5 different types were detected for *S. schenckii s. str.* and *S. globosa*, respectively. The majority of haplotypes (Hd = 0.85) belonged to *S. schenckii s. str.*, demonstrating a highly diverse group (π = 0.020). Meanwhile, *S. globosa* presented low genetic variation (Hd = 0.59; π = 0.005).

According to epidemiological data of clinical cases for the Venezuelan isolates, most of them were geographically restricted to the coastal range region (68.97%). An analysis of the distribution by gender showed a higher occurrence in male patients (64%) compared to women (36%). The most predominant clinical form in this study was fixed cutaneous (60%), followed by lymphocutaneous (36%) and one case of disseminated sporotrichosis (4%).

**Figure 3:**
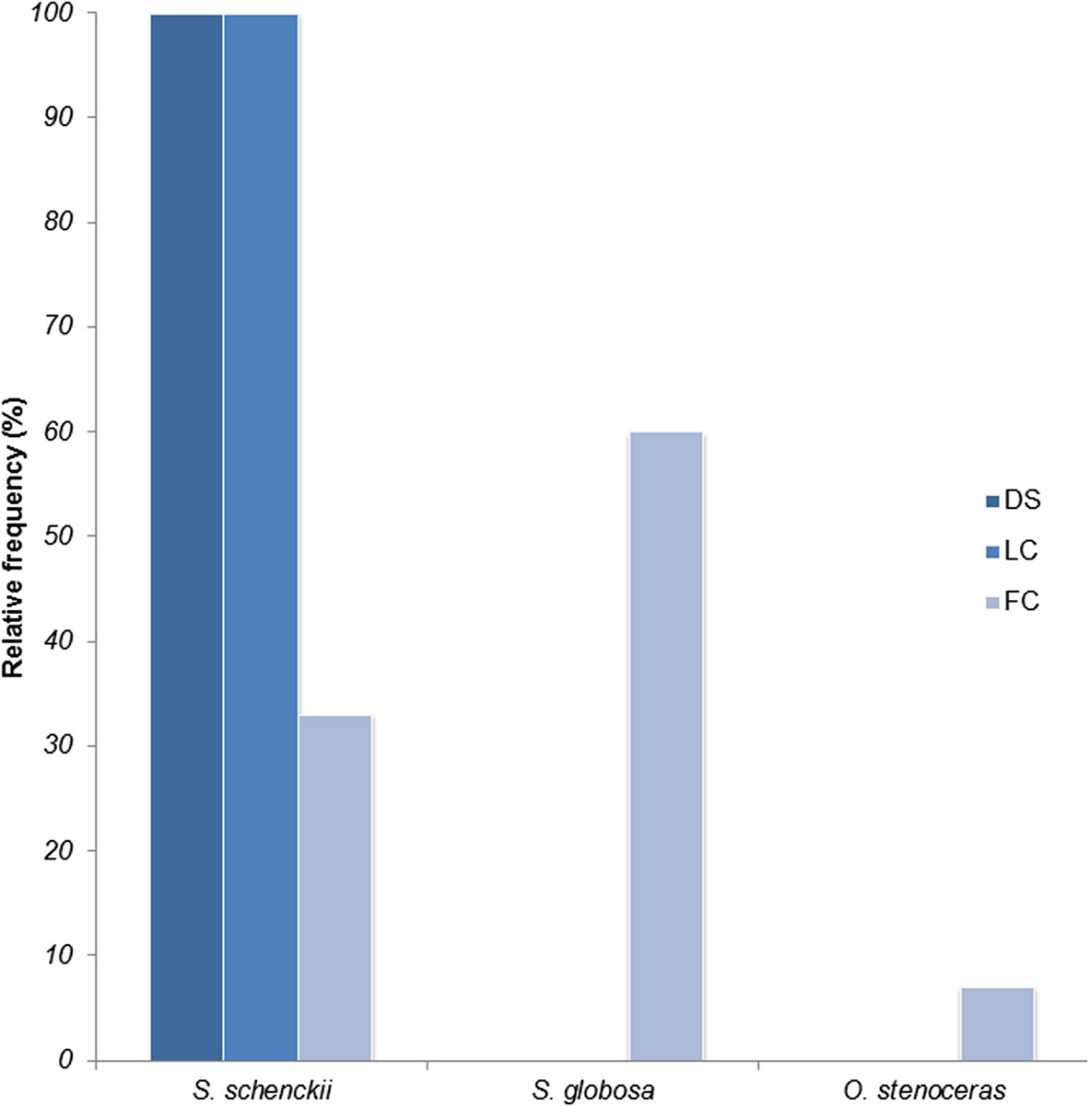
**Frequency of clinical forms of sporotrichosis vs. phylogenetic species.** DS, disseminated; LC, lymphocutaneous; FC, fixed cutaneous. Information corresponds to clinical cases with available epidemiological data (Table 1).

An association between clinical forms and phylogenetic species was found (Figure 3). All lymphocutaneous cases (100%) were caused by *S. schenckii s. str.*, while fixed cutaneous cases were attributable to both, *S. schenckii s. str.* (33.3%) and *S. globosa* (60.0%), plus a single case by *O. stenoceras*. The only case with disseminated manifestations was related to *S. schenckii s. str.*

## Discussion

Even though sporotrichosis is a cosmopolitan subcutaneous mycosis, the species grouped in the clinical clades have shown a geographical distribution [17]. *S. schenckii s. str*. presents a frequency over 50% in America, while *S. globosa* has prevailed in Asia and Europe (56% and 28%, respectively), followed by the Americas (11%) [17]. In the present study, thirty fungal isolates related to sporotrichosis in Venezuela, and previously identified by morphological studies as *S. schenckii*, were characterized by phenotypic key features and molecular phylogeny. The results show that in the country, at least two species are circulating: *S. schenckii s. str.* and *S. globosa*. Although *S. schenckii s. str.* is the main circulating species, with over 60% of evaluated cases, *S. globosa* could be considered the second most frequent species causing sporotrichosis in Venezuela. The incidence of *S. globosa*, with 30% of cases evaluated, is the highest reported so far in the Americas for this species, and is similar to its reported incidence in Europe [17]. An interesting finding is the fact that all *S. globosa* isolates here characterized, were related to fixed cutaneous sporotrichosis, which could be related to the low virulence reported for this species [33,34]. On the other hand, 100% of the lymphocutaneous cases, and the single disseminated case were all related to *S. schenckii s. str.*, as well as 33% of fixed cutaneous sporotrichosis.

In a recent study [13], the virulence profiles of eight *S. schenckii s. str.* were evaluated, and different degrees of virulence were found for this species, from high virulence to non-virulence. High genetic variation have been reported for *S. schenckii s. str.* [15,33,35]. Here, we also found high genetic variation for the Venezuelan *S. schenckii s. str.* isolates evaluated (Hd = 0.85; π = 0.020), while low genetic variation was obtained for the *S. globosa* isolates (Hd = 0.59; π = 0.005). Summing up the preceding results, we could hypothesize that a relationship might be present between the high genetic variation, different clinical presentations, and multiple virulence levels found for *S. schenckii s. str.*, while the low genetic variation of *S. globosa* isolates might be related to a single clinical presentation (fixed cutaneous sporotrichosis), and low virulence profiles related to this species [11,13]. However, in Northeast China, clinical cases of sporotrichosis (from fixed cutaneous to disseminated forms), have been attributable exclusively to *S. globosa* [36]. To confirm or discard our hypothesis, further studies are required, evaluating whether correlations can be found between virulence profiles, species, patient’s immunological conditions, and clinical presentation of isolate’s source.

Earlier characterization reports of *Sporothrix* spp. comparing the phenotypic key features for species identification proposed by Marimon *et al*. [18], and molecular phylogeny methodologies, have shown few disagreements for species identification [13,16,32,37]. In the present study, a high proportion (70%) of Venezuelan isolates related to sporotrichosis, were not identified according to their phenotype key features (as proposed by Marimon *et al*. [18]). We discarded a technical problem, since all reference strains were accurately identified by this method (Table 2). So, at least for the Venezuelan isolates here studied, the phenotypic characterization was not useful for species identification.

In our molecular phylogenetic analyses based on CAL and ITS sequences, a strong separation between clinical and environmental clades was evident (Figures 1 and 2), in correspondence with previous reports [6,16-18,33]. Isolate C8213, identified as *O. stenoceras* was isolated from an immunocompetent patient, supporting the idea that mammal-pathogenicity of Ophiostomales, outside the *S. schenckii* spp. complex, although highly exceptional [17], still can occur. To the best of our knowledge, this is the first report of *O. stenoceras* misidentified as causative agent of sporotrichosis in the Americas. Further studies into the mechanisms of opportunistic fungal pathogenesis would help to understand why a harmless fungus, as *O. stenoceras*, could become an infective agent, even for immunocompetent patients. This becomes even more relevant, when we consider the increasing number of reports of *Sporothrix* infection in immunocompromised patients, mainly the HIV-infected population [1].

Even when selection of the isolates here studied was made trying to cover a broad geographical area of the country, most isolates originated from clinical cases of the coastal region, which can be explained, besides it being a possible ecological niche for the species, to the fact that although this area covers only 3% of the total surface of the country, it is also the most heavily populated region. Further work with larger data on molecular epidemiology of sporotrichosis in Venezuela is required to verify whether correlation between clinical forms and phylogenetic species exists, and gain better knowledge about *S. schenckii* species complex spatial distribution in the country.

## Conclusions

The present study shows that sporotrichosis in Venezuela is caused at least by two species: *S. schenckii s. str.* and *S. globosa,* the latter representing the highest incidence reported in the Americas (30%). Our results firmly demonstrate that phenotypic key features for species distinction should be used with extreme caution and molecular methods should be always employed. Sporotrichosis clinical forms could be related to genetic variation of *S. schenckii* spp*.* complex and their virulence profiles. We strongly support application of ITS as a fungal barcoding gene for species distinction of all clinical and environmental clades of *Sporothrix*, and accidental infective agents within the Ophiostomales.
